# Flow Cytometry-Based Characterization of Mast Cells in Human Atherosclerosis

**DOI:** 10.3390/cells8040334

**Published:** 2019-04-09

**Authors:** Eva Kritikou, Marie A.C. Depuydt, Margreet R. de Vries, Kevin E. Mulder, Arthur M. Govaert, Marrit D. Smit, Janine van Duijn, Amanda C. Foks, Anouk Wezel, Harm J. Smeets, Bram Slütter, Paul H.A. Quax, Johan Kuiper, Ilze Bot

**Affiliations:** 1 Division of BioTherapeutics, Leiden Academic Centre for Drug Research, Leiden University, 2333 CC Leiden, The Netherlands; e.kritikou@lacdr.leidenuniv.nl (E.K.); m.a.c.depuydt@lacdr.leidenuniv.nl (M.A.C.D.); kevin.evan@hotmail.com (K.E.M.); j.van.duijn@lacdr.leidenuniv.nl (J.v.D.); a.c.foks@lacdr.leidenuniv.nl (A.C.F.); b.a.slutter@lacdr.leidenuniv.nl (B.S.); j.kuiper@lacdr.leidenuniv.nl (J.K.); 2 Department of Surgery, Leiden University Medical Center, 2333 ZC Leiden, The Netherlands; m.r.de_vries@lumc.nl (M.R.d.V.); a.m.govaert@lumc.nl (A.M.G.); m.d.smit@lumc.nl (M.D.S.); p.h.a.quax@lumc.nl (P.H.A.Q.); 3 Einthoven Laboratory for Experimental Vascular Medicine, Leiden University Medical Center, 2333 ZC Leiden, The Netherlands; 4 Department of Surgery, HMC Westeinde, 2512 VA The Hague, The Netherlands; a.wezel@haaglandenmc.nl (A.W.); h.smeets@haaglandenmc.nl (H.J.S.)

**Keywords:** atherosclerosis, mast cell, flow cytometry, tryptase, plaque stability

## Abstract

The presence of mast cells in human atherosclerotic plaques has been associated with adverse cardiovascular events. Mast cell activation, through the classical antigen sensitized-IgE binding to their characteristic Fcε-receptor, causes the release of their cytoplasmic granules. These granules are filled with neutral proteases such as tryptase, but also with histamine and pro-inflammatory mediators. Mast cells accumulate in high numbers within human atherosclerotic tissue, particularly in the shoulder region of the plaque. These findings are largely based on immunohistochemistry, which does not allow for the extensive characterization of these mast cells and of the local mast cell activation mechanisms. In this study, we thus aimed to develop a new flow-cytometry based methodology in order to analyze mast cells in human atherosclerosis. We enzymatically digested 22 human plaque samples, collected after femoral and carotid endarterectomy surgery, after which we prepared a single cell suspension for flow cytometry. We were able to identify a specific mast cell population expressing both CD117 and the FcεR, and observed that most of the intraplaque mast cells were activated based on their CD63 protein expression. Furthermore, most of the activated mast cells had IgE fragments bound on their surface, while another fraction showed IgE-independent activation. In conclusion, we are able to distinguish a clear mast cell population in human atherosclerotic plaques, and this study establishes a strong relationship between the presence of IgE and the activation of mast cells in advanced atherosclerosis. Our data pave the way for potential therapeutic intervention through targeting IgE-mediated actions in human atherosclerosis.

## 1. Introduction

Up to the present day, atherosclerosis, the main underlying pathology of acute cardiovascular syndromes like stroke or myocardial infarction, is the major cause of human mortality [1]. As in most pathological conditions, the response of the immune system is crucial in the advancement of atherosclerosis, with mast cells being key mediators in this process [2]. Mast cells are innate immune cells, unique for their notorious granular load release upon activation with antigen-sensitized IgE fragments, in allergic reactions [3]. Aside from allergic inflammation, mast cells have long been established to play an important pro-inflammatory role in the development of atherosclerosis in experimental studies, as well as in human subjects [4]. Mast cells reside at low numbers in normal arterial tissue, however, their numbers increase in the arteries where a lipid-rich atherosclerotic plaque is formed [5]. In fact, as human atherosclerosis progresses, mast cells become increasingly activated and excrete their granules in the surrounding tissue [6]. The degranulated material consists mainly of proinflammatory cytokines, histamine, and neutral proteases, such as chymase and tryptase [7]. Mast cell activation is reported to augment plaque progression [8], enhance plaque destabilization [9], and increase the levels of intraplaque hemorrhage incidence [10]. Previous attempts to characterize mast cells and their protease content in human atheromata, by the means of immunohistochemistry, have revealed that mast cells comprise a heterogeneous population; the majority of cells contain only tryptase, while a smaller proportion contains chymase and tryptase [11]. Both of these proteases have been investigated in experimental studies of atherosclerosis. In these studies, tryptase has been implicated in atherosclerotic plaque destabilization [12], while the inhibition of chymase limited atherosclerotic plaque development and progression [13].

In human plaques, mast cells have furthermore been found to correlate with typical atherogenic immune populations, such as dendritic cells and T cells [14]. Importantly, in a human study of 270 patients, intraplaque mast cells emerged as the primary immune cell type to be positively associated with future cardiovascular events [15], and may thus actively contribute to atherosclerotic plaque destabilization. Until this day however, the means by which mast cells get activated inside atherosclerotic plaques have not been elucidated in full detail. However, it is suggested that the classical IgE-sensitized pathway [16,17] is involved to a certain extent. Moreover, there is increasing in vivo evidence that additional atherosclerosis-specific mechanisms can trigger mast cell activation, independently of IgE-binding [18,19], such as activation through Toll-like receptors (TLRs) [20], complement receptors [21], or neuropeptide [22] receptors. Thus far however, the proportional effect of these distinct pathways involved in atherosclerosis has not been clarified.

The presence of mast cells has thus far only been established by means of immunohistochemical staining. While this provides important information regarding the location of mast cells in the lesion, this method is limited by the number of markers that can be used to identify a cell type and its activation status. To be able to characterize the mast cell population in human atherosclerotic lesions in more detail using multiple markers simultaneously, we aimed to develop a novel flow cytometry-based technique to identify the mast cell population in human atherosclerosis, and to determine its activation status.

## 2. Methods

### 2.1. Sample Collection and Processing

The atherosclerotic plaque material of 22 anonymous human subjects was collected peri-operatively from carotid (*n* = 10) and femoral (*n* = 12) artery endarterectomy (from July to December 2016 at the Haaglanden Medical Center Westeinde, The Hague, The Netherlands). The handling of all of the human samples complied with the “Code for Proper Secondary Use of Human Tissue”, METC number 16-071. The plaque samples were placed in RPMI (Lonza, Breda, The Netherlands) directly after removal from the patient. The culprit part of the plaques was collected as described previously [23], and stored in Shandon Zinc Formal-Fixx (Thermo Scientific, Waltham, MA, USA) for histology purposes. The remainder (~90%) of the plaques were processed into single cell suspensions by a 2-h digestion step in 37 °C, with an enzyme mix consisting of collagenase IV (Thermo Scientific, Waltham, MA, USA) and DNase (Sigma, Zwijndrecht, The Netherlands), as previously described [24]. Subsequently, the samples were filtered through a 70 μm cell strainer to obtain single cells, which were kept in RPMI/1% Fetal Calf Serum (FCS) until further analysis.

### 2.2. Histology

The culprit part of atherosclerotic samples was placed in Kristensen’s buffer for three to seven days for decalcification, after which the plaques were embedded in paraffin. Next, the plaques were sectioned in 5-µm thick sections using a microtome RM2235 (LEICA Biosystems, Amsterdam, The Netherlands). A Movat’s pentachrome staining was routinely performed, and subsequently, the plaques were analyzed for histological parameters, as described in Table 1 (three sections/plaque), based on the semiquantitative scoring systems of the AtheroExpress biobank [23] and the Oxford Plaque Study [25]. In short, the plaques were assessed for the presence of unstable plaque features such as the presence of a necrotic core, inflammatory cells, and intraplaque hemorrhage, as well as stable plaque features such as smooth muscle cell (SMC)-rich extracellular matrix (ECM). To identify the mast cells in the lesion, atherosclerotic plaque sections were immunohistochemically stained for tryptase using an alkaline phosphatase-conjugated antibody directed against tryptase (1:250, clone G3, Sigma, Zwijndrecht, The Netherlands), after which nitro-blue tetrazolium and 5-bromo-4-chloro-3’-indolyphosphate were used as a substrate. Nuclear Fast Red was used as a counterstaining for the nuclei. For the morphologic analysis, slides were analyzed using a Leica DM-RE microscope (Leica Ltd., Cambridge, UK).

### 2.3. Flow Cytometry

The single plaque cells were stained with extracellular antibodies containing a fluorescent label, or were fixated and permeabilized (BD Biosciences, San Jose, CA, USA) for intracellular staining (Table 2). The fluorescently labeled samples were measured on a FACS Canto II (BD Biosciences, San Jose (CA), USA) or Cytoflex (Beckman Coulter, Miami, FL, USA), and were analyzed using FlowJo software (v10).

### 2.4. Statistics

All of the data are depicted using GraphPad Prism 7.00. The values were tested for normalcy. Upon non-Gaussian distribution, an unpaired Mann–Whitney *U*-test was performed. In the case of more than two groups, a one way-ANOVA analysis was used. Differences lower than *p* < 0.05 were considered statistically significant.

## 3. Results

**Figure 1:**
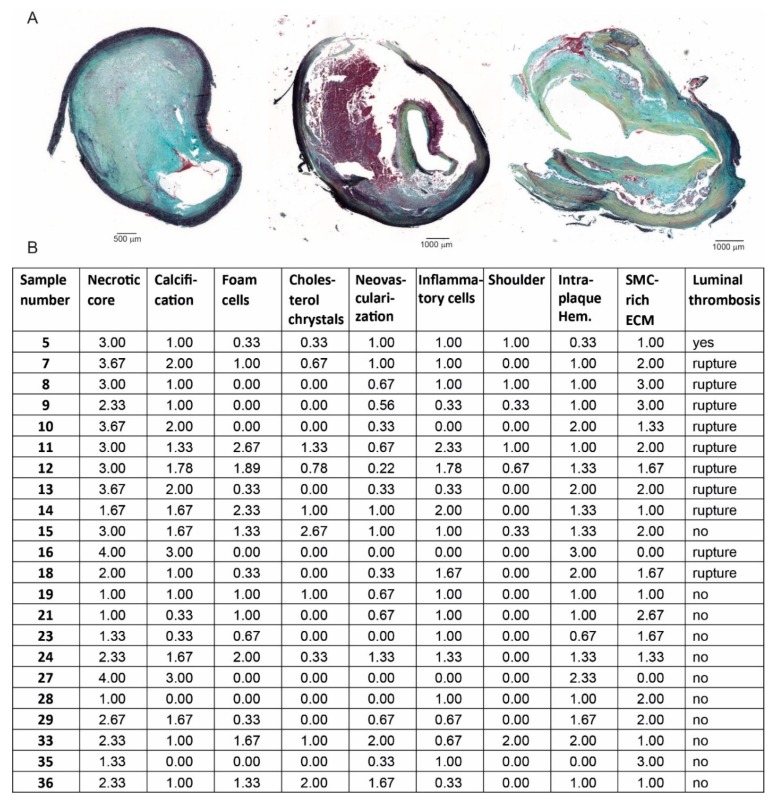
Human plaque characteristics. (**A**) Examples of Movat’s pentachrome stained human endarterectomy plaques. (**B**) Assessment of the plaque stability parameters of the individual plaques used for mast cell flow cytometry. SMC—smooth muscle cell; ECM—extracellular matrix.

We analyzed the culprit part of the carotid and femoral plaques for its histology characteristics, based on the Movat’s pentachrome staining (Figure 1A). The characteristics of the individual plaques and the assessment of the plaque stability parameters are shown in Figure 1B. Overall, the presence of a necrotic core, inflammatory cells, and intraplaque hemorrhage establish that the majority of the plaques can be classified as advanced, as expected.

**Figure 2:**
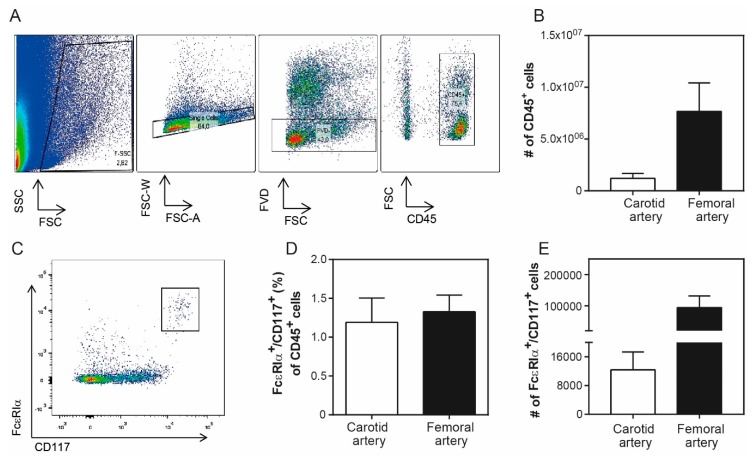
Mast cell content in human plaques. (**A**) Human plaque cell gating strategy using flow cytometry. Human intraplaque cells were selected based on their size and area. The viable cells were further separated according to the negative incorporation of fluorescent viability dye (FVD^−^). Immune cells were detected using the pan-leukocyte marker CD45^+^. (**B**) Both the femoral and carotid artery plaques contain CD45^+^ immune cells. (**C**) The human mast cell population was further classified using antibodies against the characteristic markers FcεRIα^+^ and CD117^+^. (**D**) Mast cell percentage inside human plaques isolated upon endarterectomy surgeries in carotid and femoral arteries. (**E**) Absolute mast cell numbers of human carotid and femoral artery samples. The data are depicted as mean ± standard error of the mean (SEM); (*n* = 10–12/grp).

Next, we prepared single cell suspensions of the remainder of the individual plaques, and stained the cells for the surface markers to be analyzed using flow cytometry. In Figure 2A, we demonstrate the gating strategy that we followed in order to detect the human intraplaque immune cells. Specifically, we pre-selected all of the cells from the debris present in the human plaques based on their size (forward scatter, FSC) and granularity (side scatter, SSC). Of these, single cells were further separated according to their width (FSC-W) and area (FSC-A). In addition, the viability was detected according to the negative signal for a fluorescent viability dye (FVD^−^). Viable white blood cells were identified according to the expression of the pan-leukocyte marker CD45. As the femoral plaques were generally bigger in size upon surgical removal compared with the carotid plaques, we were able to isolate more CD45^+^ immune cells from the femoral arteries (carotid: 12 × 10^5^ ± 4.7 × 10^5^ leukocytes vs. femoral: 76 × 10^5^ ± 27 × 10^5^ leukocytes; Figure 2B).

Within these cells, we detected the population of intraplaque mast cells based on the high expression of their characteristic markers FcεRIα, the receptor for IgE [26], and of CD117—the receptor for stem cell factor, a growth factor required for the end-stage maturation of mast cells [27] (Figure 2C). Accordingly, we observed that the percentage of mast cells out of all of the viable leukocytes present inside the atherosclerotic plaques is 1.19% ± 0.31% for the carotid arteries (*n* = 9), and 1.32% ± 0.21% for the femoral arteries (*n* = 13) (Figure 2D). Of note, a number of leukocytes may have been retained in the excluded debris material. Nonetheless, we enumerated the viable cells after manual quantification using Trypan Blue in relation to the percentage of viable CD45^+^ cells observed according to our gating strategy. We detected that the carotid plaques consisted of a mean 12 ± 5 × 10^3^ mast cells, while the femoral plaques contained 94 ± 37 × 10^3^ mast cells. Thus, because of the higher total number of leukocytes, we identified higher absolute mast cell numbers in the femoral plaques compared with the carotid plaques (Figure 2E).

**Figure 3:**
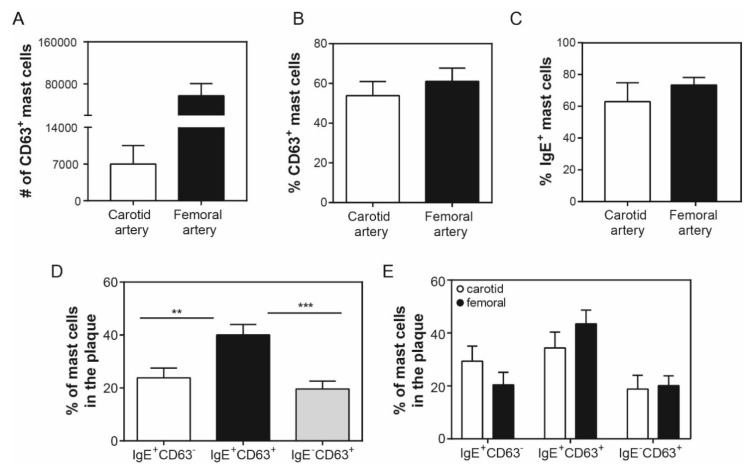
Activation of human intraplaque mast cells. (**A**) Number of activated mast cells, as defined by marker CD63, inside the carotid and femoral artery human plaques. (**B**) Percentage of activated mast cells in human atherosclerotic plaques. (**C**) Percentage of IgE^+^ mast cells in human atherosclerotic plaques. (**D**) Percentage of IgE^+^CD63^−^, IgE^+^CD63^+^, and of IgE^−^CD63^+^ mast cells of both carotid and femoral human atherosclerotic plaques combined. (**E**) Percentage of IgE^+^CD63^−^, IgE^+^CD63^+^, and of IgE^−^CD63^+^ mast cells displayed separately for carotid and femoral arteries, showing a similar pattern per plaque location. Data are depicted as mean ± SEM; A, B, C, and E: *n* = 10–12/grp; D: *n* = 22.

We proceeded to further characterize the human intraplaque cells with respect to their activation status. We therefore screened our cells for the expression of CD63, a lysosomal protein that fuses with the membrane upon the release of cellular content, and marks mast cell activation [28]. We detected 7.0 ± 3.5 × 10^3^ CD63^+^ mast cells in carotid plaques, which indicates that about 56% of these cells are in an activated state, while the femoral plaques showed 57 ± 23 × 10^3^ activated mast cells, at a total percentage of 61% (Figure 3A,B). As the mast cell activation in, for example allergic reactions, predominantly occurs via IgE bound to its receptor (FcεR), we also stained the cells for the levels of IgE bound on their surface, and observed that inside the carotid arteries 7.8 ± 3.4 × 10^3^ intraplaque mast cells, or approximately 63% of the total mast cell population, contained IgE, whereas in the femoral plaques 77 ± 36 × 10^3^ mast cells, or 74% of all of the mast cells had IgE on their surface. Because IgE binding generally implies mast cell degranulation, we analyzed, within each arterial plaque sample, the population of mast cells that showed both bound IgE and CD63 expression (IgE^+^CD63^+^), as opposed to only IgE-binding (IgE^+^CD63^−^) and only a CD63 expression (IgE^−^CD63^+^) (Figure 3B). We observed that 23.8% ± 3.7% of all human plaque mast cells have IgE bound on their surface without expressing CD63, whereas the majority of mast cells, with 40.0% ± 3.9%, appeared to have IgE bound on their surface, and had also undergone degranulation. Interestingly, a proportion of mast cells, namely 19.6% ± 2.9%, appeared to be activated without showing any IgE-fragments bound on their surface, suggesting that this mast cell fraction had been activated via alternative mast cell activation pathways. The IgE-activated population was however significantly higher than the cells subjected to non-IgE mediated activation (*p* = 0.0005), and also higher than the mast cell population that showed a binding of IgE without being activated (*p* = 0.0067). When analyzing the carotid and femoral arteries separately, similar expression patterns were observed (Figure 3E), suggesting that intraplaque mast cell activation mechanisms do not differ much between plaque locations.

**Figure 4:**
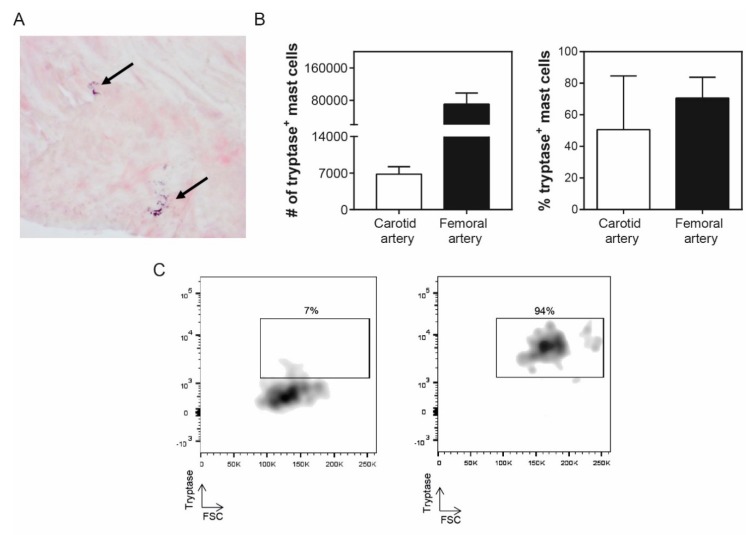
Tryptase content of mast cells in human atherosclerotic plaques. (**A**) Immunohistochemical tryptase staining of a human atherosclerotic plaque. Arrows indicate activated mast cells. (**B**) Number (left panel) and percentage (right panel) of mast cells containing tryptase in human carotid and femoral arteries based on flow cytometry. (**C**) Flow cytometry plot examples of tryptase^+^ mast cells, that is, left panel: low tryptase expression (7% of the mast cell population) vs. right panel: high tryptase expression (94% of the mast cell population). Values are depicted as mean ± SEM.

As tryptase is the preferred marker to determine the presence of mast cells in tissue by means of immunohistochemistry (Figure 4A), we also quantified the CD117^+^FcεRI^+^ mast cell populations that contained tryptase in a smaller sample of the same patients using flow cytometry (*n* = 7). We determined that 6.8 ± 1.4 × 10^3^ of the carotid intraplaque mast cells, and 71 ± 27 × 10^3^ of the femoral intraplaque mast cells were stained positive for tryptase (Figure 4B). In Figure 4C, a flow cytometry plot of an atherosclerotic plaque with a low number of tryptase^+^ mast cells (left panel) and one with a very high number of tryptase^+^ mast cells (right panel) are shown. As not all intraplaque mast cells seem to stain positive for tryptase, these data indicate that one may underestimate the number of mast cells using immunohistochemistry. In addition, the amount of tryptase^+^ mast cells in the plaque can apparently differ between patients, which may be caused by recent degranulation of the intraplaque mast cells. However, this remains to be further investigated in larger patient populations.

## 4. Discussion

In this study, we provide a novel flow cytometry-based approach to identify and characterize the mast cells in human atherosclerotic plaques. We classified ~1% of the total CD45^+^ leukocyte population obtained from the plaque tissue as mast cells, based on the CD117 and FcεRI expression. Previous data have established that these mast cells, although present in relatively low numbers in the plaque, can have a severe impact on plaque stability and on the risk of future cardiovascular events [15]. The majority of the mast cells present in arteries with advanced atherosclerosis are activated through IgE-binding, while a smaller fraction can undergo non-IgE-dependent activation. While IgE levels in the circulation [17] have been linked to increased incidence of acute cardiovascular events, and IgE fragments have been reported inside human atheromatic tissue [16], up until now, it was not clear to what extent this pathway affects mast cell activation in the area. Our data confirm that most mast cells present in the atherosclerotic plaques are activated [5,10], as it has been shown previously, specifically, for the shoulder region. We show here that the main activating mechanism is through classical antigen-sensitized IgE binding on their surface Fcε-receptors. In addition, we show that a small proportion of cells bind IgE without undergoing activation. The circulating IgE can thus bind on the surface of the intraplaque mast cells and sensitize them prior to antigen binding. The exact antigenic fragment that may cause intraplaque mast cell activation has not yet been identified, but the binding of lipid-specific antigenic fragments may be a possible mechanism [20]. Furthermore, the detection of IgE fragments inside the plaque tissue confirms that these fragments can surpass the endothelial wall and accumulate in the plaque area, which may explain why circulating IgE levels correlate with end-stage cardiovascular events like atherothrombosis [29] and myocardial infarction [30]. Therefore, it is reasonable to acknowledge IgE as an important risk factor in cardiovascular episodes, even though it still remains to be elucidated whether it is a causative element [31]. In addition, our data raise an interesting question regarding patients who suffer from other syndromes with increased circulating IgE levels. The development of atherosclerosis is a chronic process, which spans from the formation of a fatty streak, during an individual’s teenage years, and may result in an unfortunate acute event of an end-stage plaque rupture and vessel occlusion [32]. In the course of those years, a fraction of humans may be diagnosed with allergic inflammatory conditions, associating with high levels of circulating IgE [33]. This may mean that IgE has an increased chance to migrate through the endothelium and bind the intraplaque mast cells, raising the likelihood for a future cardiovascular event. In fact, there is compiling evidence that allergic asthma and atherosclerosis are linked [34], and mast cells are seemingly paramount in this [33]. In addition, patients with a genetic condition called hyper-IgE syndrome have recently been demonstrated to show signs of subclinical coronary atherosclerosis [35], and patients with systemic mastocytosis were recently described with a higher prevalence of cardiovascular disease events as compared to controls [36]. Interestingly, our group has demonstrated that the mast cell stabilizer cromolyn acts in a protective manner in atherosclerosis experimental studies [37]. Therefore, a mast cell stabilization approach may be an interesting preventive strategy in individuals who show high circulating IgE levels, but who have not yet been diagnosed with cardiovascular disease. In addition, we identified a group of mast cells that are activated without IgE binding, which suggests that this activation pathway is not the only way by which mast cells are activated in human atherosclerosis. We have previously established, in experimental atherosclerosis models, that mast cells in the vessel wall can be activated via neuropeptides such as Substance P [22] or Neuropeptide Y [38], or via complement components such as C5a [21]. These factors have also been shown to be present in human lesions [21,22]. It however remains to be established which of its receptors may induce mast cell activation in human atherosclerosis. The technical approach described in this study may be able to provide answers to these questions, by, for example, analyzing the expression of specific activating receptors on the intraplaque mast cells. It is still unclear what this implies in terms of mediator release. It would be interesting to further characterize the intracellular content of these cells to examine whether different activation pathways lead to the release of different proteases and cytokines, and how this may possibly affect the surrounding environment. Using this technique on a larger patient cohort may also reveal whether mast cell activation is affected by gender. A larger patient population will also allow for determining whether mast cell numbers or activation status relate to specific atherosclerotic plaque characteristics.

Our data provide an additional important point of attention when using tryptase-based immunohistochemistry to identify mast cells in tissue. Our flow cytometry data provide evidence that not all mast cells stain positive for tryptase, which can be explained by the fact that the recently degranulated mast cells may have released their tryptase content. One should therefore keep in mind that when using tryptase-immunohistochemistry only, the number of (activated) mast cells in the tissue may be an underestimation, and one may opt for a second quantification method, for example, a flow cytometry-based method using multiple markers.

In conclusion, in this study, we made use of flow cytometry to characterize mast cells in the advanced atherosclerotic plaques of human subjects. We confirm that the major pathway for the activation of mast cells inside the plaque tissue is IgE-mediated, and that the intraplaque mast cells are highly activated. This is particularly important, as it suggests that already available modes of therapy like mast cell stabilization agents [39] or anti-IgE treatment [40] may prove beneficial in patients suffering from atherosclerosis. We expect that larger scale patient studies and the analysis of more characterization markers, for instance through mass cytometry, will reveal new pathways via which mast cells may act in atherosclerosis, opening new ways to intervene in cardiovascular disease.

## Figures and Tables

**Table 1:** Semiquantitative grading scale for the histology score of human endarterectomy specimen.

Grade	0	1	2	3	4
**Necrotic Core**	No sign of necrotic core	<20% of the arterial area	20–40% of the arterial area	40–70% of the arterial area	>70%
**Calcification**	No sign of calcification	<10% of the arterial area	10–40% of the arterial area	>40% of the arterial area	
**Foam Cells**	No sign of foam cells	Small * A few small sized clusters (~5) of foam cells	Intermediate * Multiple small (~5) or medium sized (~10) clusters of foam cells	Large * At least 1 large sized cluster (~15) of foam cells or foam cells scattered around ~70% of the arterial area	
**Cholesterol Crystal**	No sign of cholesterol crystal	<10% of the necrotic core	10–40% of the necrotic core	>40% of the necrotic core	
**Neovasculari-zation**	No sign of neovasculari-zation	<25 neovessels in the whole tissue	25–50 neovessels in whole tissue	>50 neovessels in the whole tissue	
**Inflammatory Cells**	No sign of inflammatory cells	Small * A few (1 or 2) small sized (~50 cells) clusters of inflammatory cells or a few cells occasionally spread throughout the arterial area	Intermediate * Multiple (~5) small sized (~50 cells) clusters of inflammatory cells	Large * At least 1 large sized (~100 cells) cluster of inflammatory cells or cells scattered around ~70% of the arterial area	
**Shoulder**	Not detectable *	No sign of shoulder regions	One sided shoulder region	Two-sided shoulder region	
**Intraplaque hemorrhage (IPH)**	No sign of IPH	<10% IPH of the arterial area	10–40% IPH of the arterial area	>40% IPH of the arterial area	
**SMC-rich ECM area of total ECM area**	No SMC visible	<20% of the ECM area	20–40% of the ECM area	>40% of the ECM area	
**Luminal thrombosis**	No	Yes (rupture)			

SMC—smooth muscle cell; ECM—extracellular matrix.

**Table 2:** List of extracellular and *intracellular* antibodies used.

Antibody	Fluorochrome	Clone	Concentration	Company
Fixable Viability Dye	eFluor 780	-	0.1 μg/sample	eBioscience
CD45	PB	2D1	0.25 μg/sample	eBioscience
FcεRIα	APC/PE Cy7	AER-37	0.12 μg/sample	eBioscience
CD117	PercP Cy5.5	104D2	0.1 μg/sample	eBioscience
CD63	PE	H5C6	0.1 μg/sample	eBioscience
IgE	PE Cy7/FITC	Ige21	0.25 μg/sample	eBioscience
*Tryptase/TPSAB1*	*PE*	-	0.1 μg/sample	LSBio
